# Role of ultrasonic treatment, inoculation and solute in the grain refinement of commercial purity aluminium

**DOI:** 10.1038/s41598-017-10354-6

**Published:** 2017-08-29

**Authors:** G. Wang, Q. Wang, M. A. Easton, M. S. Dargusch, M. Qian, D. G. Eskin, D. H. StJohn

**Affiliations:** 1 Centre of Advanced Materials Processing and Manufacturing (AMPAM), The University of Queensland, St Lucia, QLD 4072 Australia; 2 Defence Materials Technology Centre (DMTC), The University of Queensland, St Lucia, QLD 4072 Australia; 3 Centre for Additive Manufacturing, School of Engineering, RMIT University, Melbourne, VIC 3000 Australia; 4 BCAST, Brunel University, London, Uxbridge UB8 3PH United Kingdom; 5 Tomsk State University, 634050 Tomsk, Russian Federation

## Abstract

The present study investigates the influence of ultrasonic treatment on the grain refinement of commercial purity aluminium with a range of Al3Ti1B master alloy additions. When the aluminium contains the smallest amount of added master alloy, ultrasonics caused significant additional grain refinement compared to that provided by the master alloy alone. However, the influence of ultrasonics on grain size reduces with increasing addition of the master alloy which adds additional TiB_2_ particles and Ti solute with each incremental addition. Applying the Interdependence model to analyse the experimentally measured grain sizes revealed that the results of this study and those from similar experiments on an Al-2Cu alloy were consistent when the alloy compositions are converted to their growth restriction factors (*Q)* and that increasing *Q* had a major effect on reducing grain size and increasing grain number density. Compared with the application of ultrasonic treatment where an order of magnitude increase in the grain number density is achieved, an increase in the Ti content over the range of master alloy additions, causes the grain number density to increase by approximately three times.

## Introduction

Many techniques have been used to refine the as-cast grain structure of metals, such as inoculation by adding a grain refining master alloy into the melt^1–3^ and changing local nucleation and growth conditions by external forces^4–7^. The addition of master alloys containing inoculant particles is standard grain-refining practice for many aluminium alloys where the most popular grain refiners are Al-Ti-B and Al-Ti-C master alloys^8, 9^. The application of an external field such as Ultrasonic Treatment (UST) has proved to be effective for the grain refinement of various Al alloys as has been well documented and reviewed^4, 10–15^.

It has been shown that UST can affect the efficiency of refinement when applied in the fully liquid state, during the manufacture of master alloys and during the nucleation stage of solidification. The improved efficiency of grain refining was previously shown for 2XXX-series alloys upon UST of the melt in the launder on the way to the mould combined with the introduction of an Al5Ti1B grain refining rod^4, 14^. Deagglomeration of the boride particles along with their better distribution was suggested as the reason for the improved efficiency. A number of researchers have further investigated the effect of UST on the microstructure of Al-Ti-B and Al-Ti-C master alloys during the fabrication of the master alloys^16–18^. It was revealed that the reaction among halide salts and aluminium is accelerated when the melt is treated with ultrasound, and not only the morphology of TiAl_3_ phase is improved, but also the boride particles within agglomerations take on a spawn-like form which further improves the performance of the master alloy^16^. A comparison study on the grain refinement of pure aluminium through either UST applied during solidification or the addition of a master alloy Al-Ti-B has shown that the grain size in the pure aluminium ingots subjected to ultrasonic vibrations was comparable to those with the addition of grain refiners^19, 20^.

It is well accepted that the growth restriction factor, Q, of an alloy plays an important role in defining the grain structure^21, 22^. Experimental results from ultrasonic grain refinement of Al–Zr–Ti alloys reveals that when the concentration of Zr is sufficient to produce primary intermetallics and a small amount of Ti is present in the melt, then ultrasonic processing results in very strong grain refinement^11, 22^. It is believed that the increased potency of the primary particles of the ultrasonic treatment contributes to the grain refinement along with hindering the growth of the grains by the high growth restricting Ti solute present in the melt. The grain size was related to the inverse growth restriction factor, 1/Q. A similar relationship was found for Mg-Zn and Mg-Al alloys either without or with UST^23–25^. In particular, experimental results showed that increasing the solute content in both Mg-Al and Mg-Zn alloys at a low UST intensity level (e.g., 100–400 W cm^−2^ but still above the cavitation threshold) was even more effective in grain refinement than substantially increasing the UST intensity level^25^.

There is, however, limited understanding about the interactive effect of Al-Ti-B master alloys and UST on the grain refinement of aluminium and aluminium alloys^4, 11^. The present research investigates the combined effect of Al3Ti1B master alloy in terms of both inoculation and solute and UST on the grain refinement of commercial purity aluminium (CP Al). The grain refining efficiency is evaluated for a range of master alloy additions with and without the application of UST, compared to results from similar experiments on an Al-2 wt% Cu alloy (Al-2Cu)^26^ through consideration of changes in the growth restriction factor, and discussed in terms of nucleation and growth mechanisms.

## Results

### Influence of UST on Grain Refinement of CP Al

**Figure 1:**
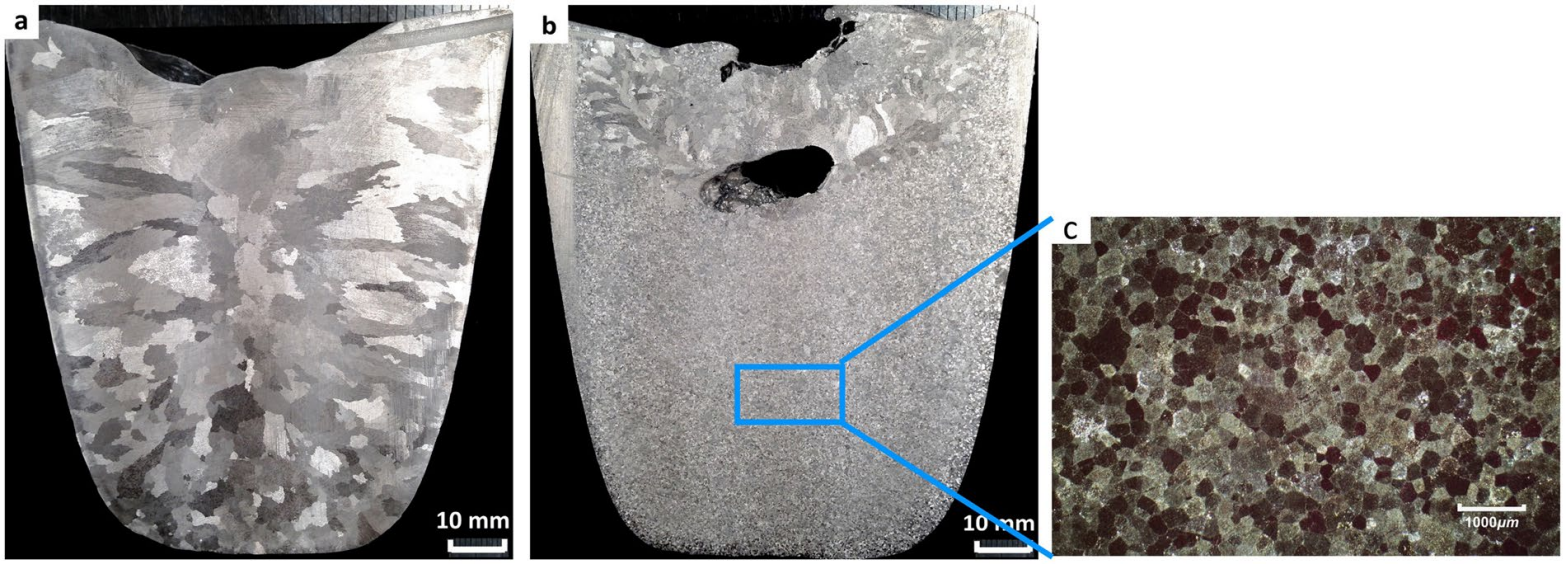
The macrostructure of the cast CP Al ingots (**a**) without UST and (**b**) with UST applied from 40 °C above the liquidus temperature for 4 minutes during cooling and solidification; (**c**) the microstructure of the centre of the UST casting as indicated in (**b**).

Solidification of the CP Al without UST and without the addition of master alloy produces a coarse columnar dendritic macrostructure (Fig. 1a). When UST is applied from 40 °C above the liquidus temperature and terminated after 4 minutes of subsequent cooling and solidification at a cooling rate of 0.8–1 °C/s, an almost fully refined equiaxed grain structure is obtained. However, the top of the ingot adjacent to the sonotrode location but above the sonotrode tip has a coarse grain structure, which demonstrates that cavitation and acoustic streaming (all happening below the sonotrode) play a decisive role in grain refinement (Fig. 1b). The same observations were made for pure magnesium^25^, A356 aluminium alloy^27^, and Al-Si alloys^28, 29^ upon ultrasonic grain refienment through immersion of a sonotrode into the melt. The microstructure of the sample from the central part of the ingot subjected to UST is shown in Fig. 1c, and the average grain size is about 160 µm indicating that significant grain refinement results from the application of UST during solidification.

### Influence of Al3Ti1B Master Alloy Addition on Grain Refinement

**Figure 2:**
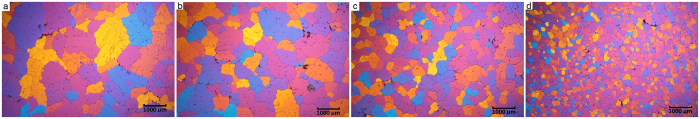
The microstructure of the samples with the addition of (**a**) 50 ppm Ti, (**b**) 100 ppm Ti, (**c**) 200 ppm Ti and (d) 1000 ppm as Al3Ti1B master alloy without UST.

Figure 2 shows the effect of adding (a) 50, (b) 100, (c) 200 and (d) 1000 ppm equivalent Ti on the microstructure near the centre of the ingot. With an increase in the addition of the grain refiner from 50 to 200 ppm, the coarse dendritic structure evolved into fine grains with a near spherical shape as shown in Fig. 2a–c. Adding 1000 ppm Ti causes a further significant change to a fine equiaxed structure as shown in Fig. 2d. The grain size was reduced from about 1300 μm to 190 μm.

### Influence of UST on the Refinement of CP Al with Master Alloy Additions

**Figure 3:**
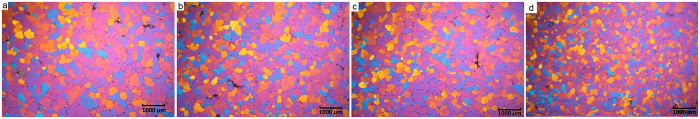
The microstructure of the samples with the addition of (**a**) 50 ppm Ti, (**b**) 100 ppm Ti, (**c**) 200 ppm Ti and (**d**) 1000 ppm as Al3Ti1B master alloy with UST.

Figure 3 presents the microstructures of the samples with (a) 50, (e) 100, (c) 200 and (d) 1000 ppm Ti after the application of UST. Figure 3a shows that the grain size is consistently smaller when UST is applied (compare with Fig. 2) and ranges from about 130 µm to just under 100 µm. It is clearly observed that for the same level of master alloy addition, the UST samples show much finer grain structures than those without UST. It is also important to note that the grain size of pure Al with grain refiner and after UST during solidification, is always smaller than the grain size after UST without grain refiner (see Fig. 1c, 160 µm).

**Figure 4:**
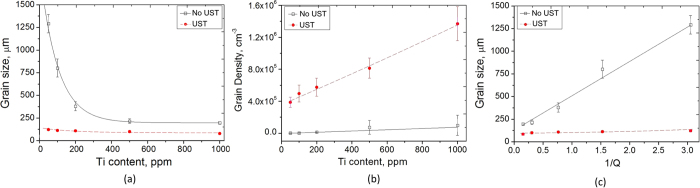
The relationships between (**a**) grain size and Ti content, (**b**) grain density and Ti content, and (**c**) grain size and the inverse growth restriction factor *Q*, for samples produced with and without the application of UST. For comparison, without addition of the master alloy the grain size is ~4000 μm (grain density 12 cm^−3^) without UST and 160 μm (grain density 2.42 × 10^5^ cm^−3^) with UST.

The average grain size of the ingot samples with and without the application of UST is plotted against the equivalent content of Ti in Fig. 4a. Without UST there is a significant decrease in grain size with an increase from 50 to 500 ppm Ti with the grain size decreasing more slowly with further increases in the Ti content. When UST is applied there is a relatively small decrease with addition level from 50 to 100 ppm Ti followed by what appears to be a near-horizontal linear relationship between Ti content and grain size. The small influence of the Ti content suggests that grain refinement in the combined case was achieved mainly by UST.

A measure of grain density is an indication of the number of nucleant particles or fragments that successfully nucleate grains assuming a 100% survival rate. Figure 4b converts the grain sizes in Fig. 4a into grain density^30^ and the linear relationships between grain density and Ti content are obtained for both cases: with and without UST. The grain density for the UST samples is significantly higher than when UST is not applied for the same level of Ti addition, which confirms that UST has a large effect on the number of nucleation events, particularly at the high Ti contents, where the difference in grain size actually appears to be smaller. The reason for this apparent discrepancy is that there is an inverse cube root relationship between grain number density and grain size (see equation 2), and as the grain size decreases the differences in grain density become larger. The much steeper slope corresponding to UST reveals that there is a significant increase in the number of grains formed (approximately ten times by comparing the slopes of two lines in Fig. 4b).

Although Fig. 4a suggests that higher Ti contents do not have a major effect on grain size when UST is applied, Fig. 4b clearly demonstrates that the Ti concentration has a significant effect on the grain density which, in the UST case, increases by more than three times as the Ti concentration is increased. Thus, both UST and Ti concentration play a significant role when applied together.

Further insight into the mechanisms of the observed effects can be gained by plotting grain size against the growth restriction factor *Q*. *Q* is equal to *mC*_0_(*k*−1) where *m* is the slope of the liquidus line, *k* is the partition coefficient *C*_s_ */C*_l_ and *C*_0_ is the alloy composition. When AlTiB master alloys are added, the Ti is present both as TiB_2_ particles and as solute in the melt. For the calculation of *Q* only the Ti in solution is used as the Ti within the TiB_2_ particles does not contribute to *Q*. The relationships between grain size and 1/*Q* with and without UST are presented in Fig. 4c. Straight lines can be fitted to the grain size, *d*, versus 1/*Q* plots for both cases, with and without UST. In comparison, the line for the UST data shows a much lower slope of 10 μm·K compared with 379 μm·K when UST is not applied.

## Discussion

Previously it was shown that ultrasonic treatment (cavitation and acoustic streaming) promotes refinement and the distribution of primary intermetallics^11, 31^, deagglomeration and a more homogeneous distribution of TiB_2_ particles introduced from grain-refining master alloys creating more sites for nucleation^14, 16, 17^. In addition, the collapse of cavitation bubbles may facilitate heterogeneous nucleation due to local pressure changes^4, 10, 32^ and/or enhanced wetting of particles which may improve their efficiency for grain refinement^4^. When applied during solidification, UST may result in fragmentation of the growing grains, further contributing to the increase in grain density^10, 32, 33^. Figure 4b clearly shows that UST can dramatically increase the grain density by an order of magnitude. In the present study, the nucleant particles are assumed to be TiB_2_ from the Al3Ti1B master alloy. Note that the actual number of active particles may change with the application of UST due to their de-agglomeration and better dispersion in the melt. Dendrite fragmentation may further add to the actual grain density.

The change in slope of the grain size versus 1/*Q* plots in Fig. 4c may be caused by several factors as mentioned above. It should be noted that a comparison between cases with and without UST using 1/*Q* plots is not strictly valid as the solidification conditions, particularly due to the interaction of the cavitation zone with the solidification front, are very different. However, the effect and possibly the contribution of alloy chemistry, particle characteristics and temperature gradient to the reduction in grain size can be evaluated by using the Interdependence model defined by Equation 1^34^ which has two terms, *x*_NFZ_ and *x*_Sd_, that contribute to the final grain size.

1 $${d}_{gs}={x}_{NFZ}+{x}_{Sd}=5.6(\frac{D\cdot z{\rm{\Delta }}{T}_{n}}{vQ})+{x}_{Sd}$$

where *D* is the diffusion coefficient, *v* is the rate of growth of the solid-liquid (*S*–*L*) interface, and *z*Δ*T*_n_ is the incremental amount of undercooling required to activate the next nucleation event as the temperature gradient moves towards the thermal centre of the casting. The second term denoted *x*_Sd_ is the distance between the most potent particles in the melt^1^. Because more TiB_2_ particles are added with each increase in master alloy addition, and additional dendrite fragments may be formed upon UST, *x*_Sd_ decreases. The first term in Equation 1, *x*_NFZ_, is the size of the Nucleation-Free Zone (NFZ) where nucleation is not able to occur due to insufficient undercooling. As *Q* increases, the contribution of *x*_NFZ_ to the final grain size decreases. The gradient of the curves in Fig. 4c can be significantly influenced by changes in the size of *x*_NFZ_ surrounding each nucleated grain as the value of *Q* changes^1^. *x*_NFZ_ can be reduced by reducing Δ*T*_n_ (i.e. increasing the potency of the particles), decreasing *z* which is related to the temperature gradient in front of a growing grain, or increasing *Q* and *v*. The range of Δ*T*_n_ is assumed to be constant for the TiB_2_ particles (providing that UST does not change their potency) and only the number density of particles change when master alloy is added or UST is applied, which affects *x*_Sd_. The variables are the range of *Q* values provided by the solute Ti and the value of *z* which may be reduced by acoustic streaming when UST is applied.

**Figure 5:**
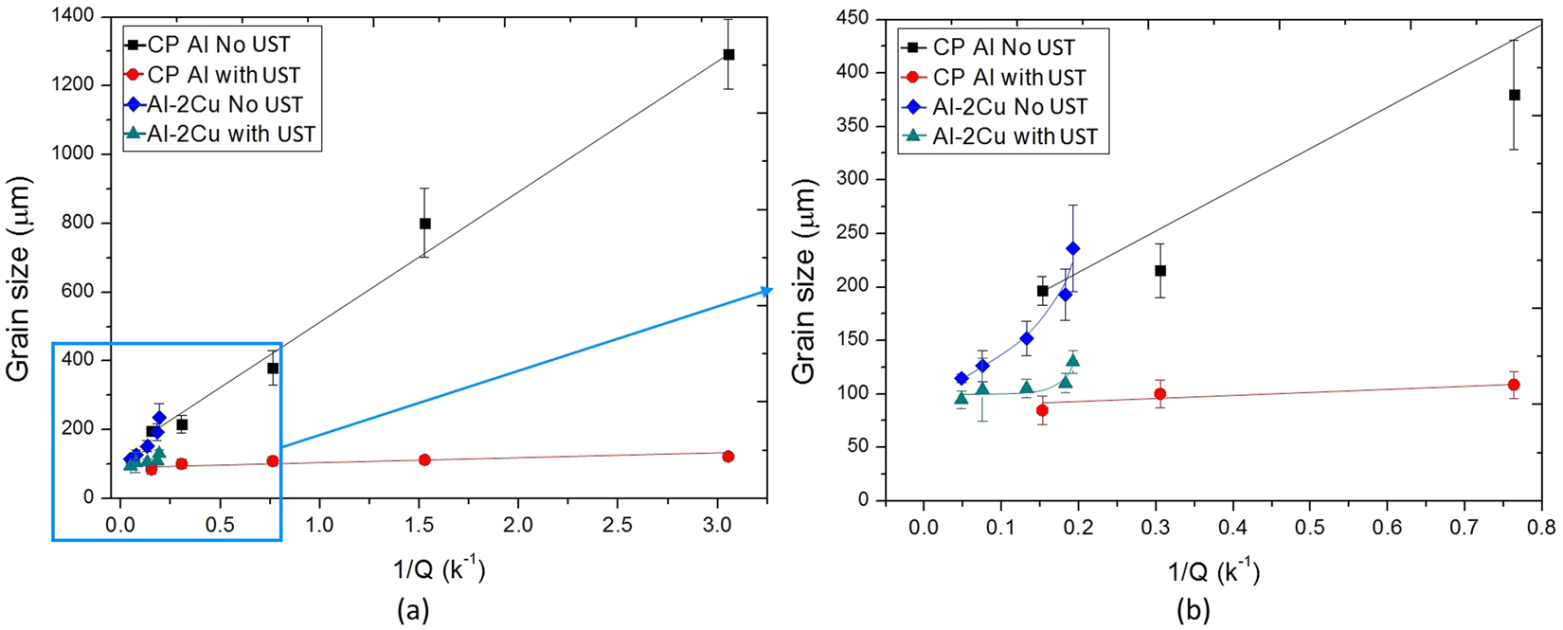
(**a**) Grain size versus 1/*Q* for CP Al and Al-2Cu; and (**b**) the same plot focused on low 1/*Q* values from 0 to 0.8.

The following analysis benefits from including grain size data from our previous work^26^ where a similar range of Al3Ti1B master alloy additions were made to an Al-2Cu alloy also treated with UST using a similar processing schedule. Table 1 presents the solute compositions of the alloys and their respective *Q* values. The addition of 2% Cu provides additional growth restriction with *Q* equal to 4.84 as derived from Thermo-Calc, which Table 1 shows is equivalent to an addition of Ti between 500 to 1000 ppm without Cu being present. This addition of 2% Cu significantly reduces the 1/*Q* values and shifts the Al-2Cu curves to lower *1/Q* values as observed in Fig. 5.

**Table 1:** *Q* values for both CP Al and Al-2%Cu alloy with the addition of Al3Ti1B^26^.

Ti content (PPM)	Ti in TiB2 (wt.%)	Ti solute (wt.%)	Q (CP Al)	Q (Al-2% Cu)
0	0	0	0	4.84
50	0.0037	0.0013	0.33	5.2
100	0.0073	0.0027	0.65	5.48
200	0.0147	0.0053	1.31	6.14
500	0.037	0.013	3.27	7.56
1000	0.073	0.027	6.55	13.28
2000	0.147	0.053	13.10	20.88

At first glance the shape of the curves for Al-2Cu and CP Al appears to be quite different. For the Al-2Cu-based alloys the combined growth restriction from both Ti and Cu results in an almost linear relationship between the grain size and 1/*Q* except for a steeper increase in grain size at the larger 1/*Q* values. On the other hand, Ti addition without the presence of Cu appears to produce a linear relationship. The grain size versus 1/*Q* relationship when UST is applied reduces the grain size to a similar value for both alloys suggesting UST has a dominant effect over grain refiner addition.

In Fig. 5b upward curvature is observed over the range of higher 1/*Q* values for the Al-2% Cu alloys. Equation 2 was developed to predict the effect on grain size of adding Ti solute and TiB_2_ particles simultaneously^34^.

2 $${d}_{{\rm{gs}}}=\mathrm{5.6}(\frac{Dz\Delta {T}_{{\rm{n}}}}{vQ})+\frac{\mathrm{1000}}{\sqrt[3]{\frac{Ti{B}_{2A}}{Ti{B}_{2MA}}\cdot {N}_{v({d}_{pi}\le {d}_{p}\le {d}_{pj})}}}$$

where *TiB*_*2A*_ is the amount of TiB_2_ added, *TiB*_*2MA*_ is the weight percent of TiB_2_ in the master alloy, *N*_*v*_ is number density of TiB_2_ particles in the master alloy that are able to become active and which are defined by the size range between *d*_*Pi*_ and *d*_*Pj*_ (in μm). The range *d*_*Pi*_ and *d*_*Pj*_ refers to the size of particles that become active nucleants and excludes smaller particle sizes that play no role in nucleation of grains. Equation 2 has been previously quantified for a large range of Al alloys with various Ti concentrations^3^ giving

3 $${d}_{gs}=\frac{652}{Q}+\frac{32}{\sqrt[3]{[{T}_{i}{B}_{2}]}}$$

**Figure 6:**
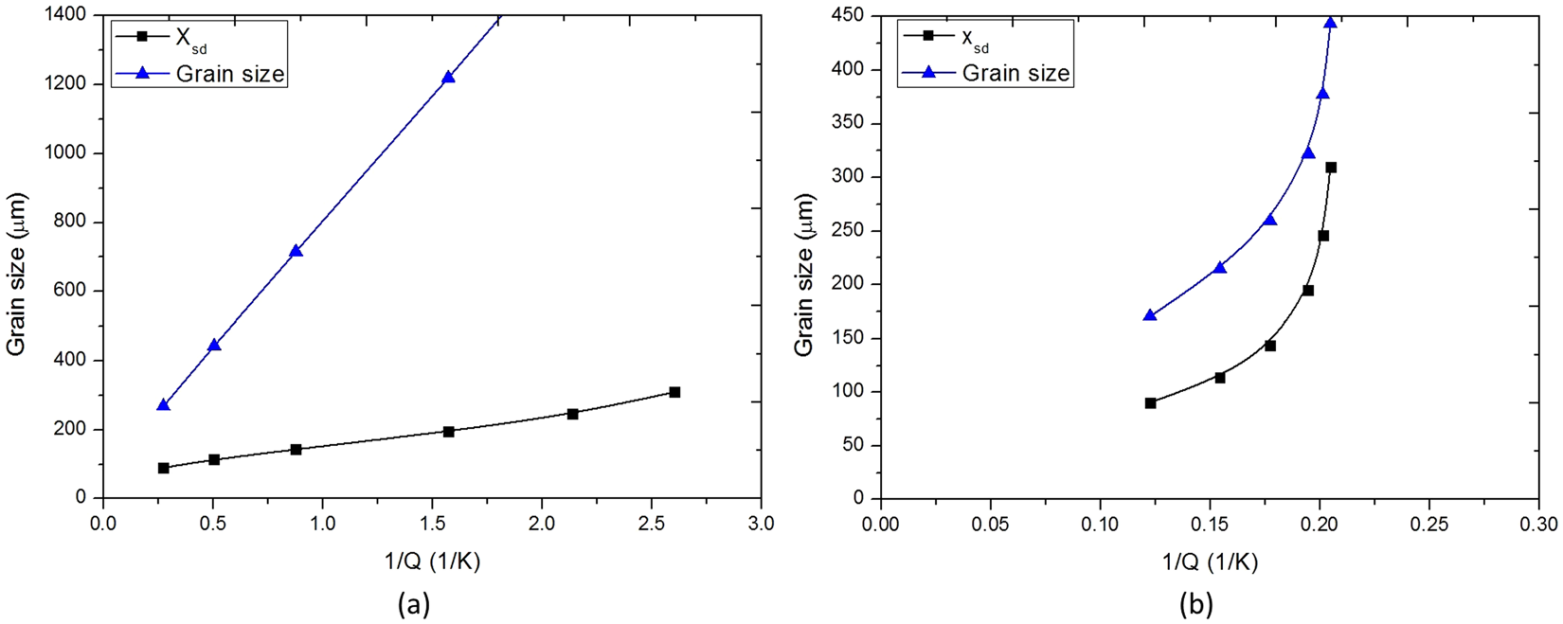
Predictions made by Eq. 3 of grain size and the contribution from *x*_Sd_ calculated by the second term in Eq. (3), for an alloy with a *Q*-value of (**a**) 0.3 typical of commercial purity Al and (**b**) 4.8 for an Al-2%Cu alloy when additions of Al3Ti1B grain refiner are made. The difference between the grain size and the *x*_Sd_ is equal to *x*_NFZ_^1^.

If the particle number density of TiB_2_ particles in the melt was constant while changing the Ti solute content a straight line plot is normally obtained^1^. However, using Equation 3, when a refiner containing TiB_2_ particles as well as Ti solute is added to alloys with relatively high values of *Q* (i.e. the Al-2Cu alloy) a steeper and curved plot is predicted (Fig. 6). On the other hand, for the refined CP Al this curvature is much shallower given the much greater range of 1/*Q*, so that a straight line gives a fit with a high correlation coefficient and can be plotted through the data, hiding any inherent curvature^35^. Additionally, when *Q* is initially very small the grain size is usually much larger with an associated large measurement error. Therefore, the approximate straight line plot observed in this study does not preclude the curvature expected from the addition of a master alloy. (It should be noted that the correlation between the grain size trends from castings without UST in Fig. 5 and the model prediction in Fig. 6 is remarkable, given that the casting environment of these experiments was somewhat different to that of the castings used to develop Equation 3. This correspondence provides some confidence regarding the following analysis.) Fig. 6 also shows the contribution of *x*_Sd_ (i.e., the second term in Eq. (3)) to the grain size. The difference between the predicted grain size and *x*_Sd_ is equal to *x*_NFZ_.

Despite the plots of grain size versus 1/*Q* (Fig. 5(b) for the Al-2Cu and CP Al refined alloys having different shapes over different ranges of *Q*, combining the data into sets with and without UST indicates a consistent relationship for each set of data. Therefore, the grain size data for both base alloys are consistent indicating that grain refiner addition and UST are affecting both alloys in a similar manner.

As mentioned above, the parameter *z* in Equation 1 represents the effect of temperature gradient on the size of *x*_NFZ_. Because the temperature gradient decreases due to convection generated by acoustic streaming during UST treatment^4, 10, 36, 37^, the value of *x*_NFZ_ reduces and, therefore, the gradient of the UST curve in Fig. 4c also reduces. For a given composition a reduction in *x*_NFZ_ enables more TiB_2_ particles to nucleate grains^1^. For example, if *x*_NFZ_ represents about two thirds of the grain size as calculated by Equation 1 (e.g. Figure 6(a)) then eliminating it would allow two more potent particles to nucleate additional grains within that zone. As Equation 1 is derived for a one-dimensional (1D) case the actual increase could be much greater, e.g., in the 3D space eight or more particles may nucleate new grains. This projected increase in particle density approaches the difference in particle number density shown in Fig. 4b.

**Figure 7:**
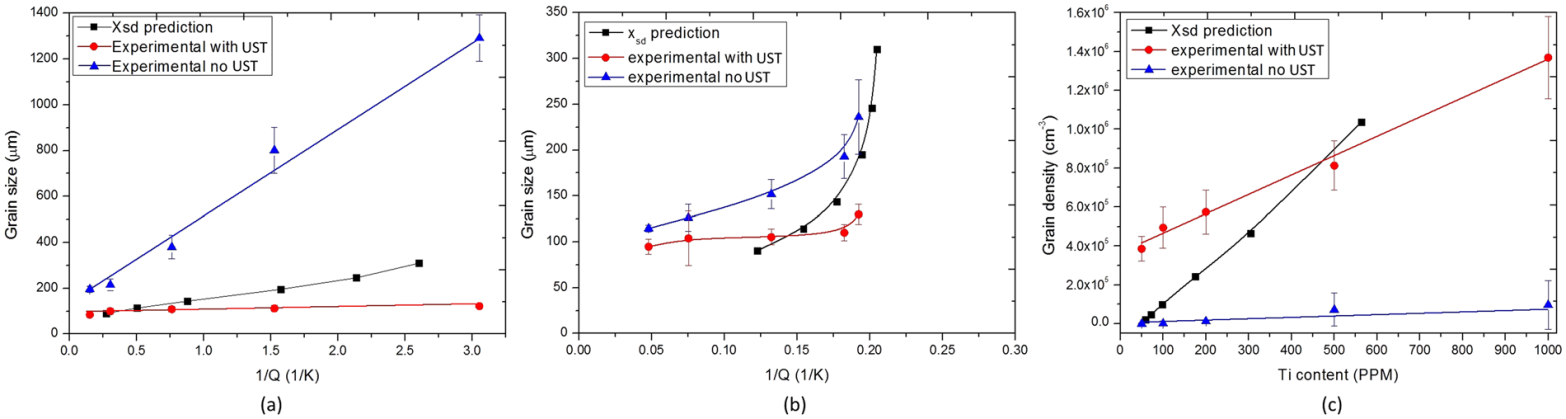
Grain size versus 1/*Q* for (**a**) CP Al and (**b**) for Al-2% Cu and (**c**) grain density in CP Al predicted by *x*_Sd_ and converted from the experimental grain size measurements with and without UST.

The *x*_Sd_ plots in Fig. 6 which are only controlled by the particle number density, predict the decrease in grain size that would be expected from eliminating *x*_NFZ_ (i.e. the first term in Equations 2 and 3). Figure 7a and b compares the *x*_Sd_ plots to the measured grain size plots with and without UST for both base alloys (from this work and from ref. 25). It can be observed that at low *Q* values (i.e. high 1/*Q*) the experimental results for UST are lower than that predicted by *x*_Sd_. At high *Q* values the two curves converge. The grain refining effect of UST is stronger than that predicted by Equation 3 at low master alloy additions (high 1/*Q*), suggesting that UST is further increasing the number of nucleation events beyond the effect of acoustic streaming on the thermal environment. Figure 7c shows the difference in grain density between that predicted by *x*_Sd_ and those converted from the experimental grain size measurements of CP Al with and without UST. At low Ti contents UST has a large additional effect on grain density. As the Ti content increases, the difference diminishes until the two curves come together at about 500 ppm Ti or when 1/*Q* is about 0.35 (Fig. 7a). There are several possible reasons for the additional nucleation events such as UST improving particle wetting and dispersion of TiB_2_ agglomerates, their subsequent distribution throughout the melt by acoustic streaming as well as dendritic fragmentation^4, 20, 36^. As a result, there may be more active substrates than assumed by Equation 3 further reducing *x*_*Sd*_. Also, UST may generate nucleation at sites independent of TiB_2_ particles due to cavitation on or near the sonotrode surface. The excellent refinement obtained when no master alloy addition is made suggests some or all of these mechanisms might be occurring.

As a result of this analysis it is proposed that when the temperature gradient is low throughout the melt due to acoustic streaming, UST can increase the number of grains formed as predicted by Equation 3 and, depending on the Ti content, result in a further increase in the number of nucleation events due to the additional ultrasonic effects particularly at the lower Ti contents. The newly formed grains are then distributed throughout the melt. UST may also affect other terms in Equation 1 such as *v* and *D* due to acoustic streaming, the degree of undercooling due to cavitation, and dendrite fragmentation. As there are a number of possible reasons for grain nucleation and multiplication, considerable further research needs to be undertaken to determine the relative contribution of each mechanism to the final as-cast grain size. Despite these considerations, the evaluation of the experimental results by the Interdependence model provides improved understanding about the relative effects of UST, master alloy content, alloy composition, and casting conditions, and the associated mechanisms operating during the development of grain size.

## Methods

1 kg of commercial purity Al ingot (99.7%) was melted in a graphite-clay crucible with 90 mm top diameter, 60 mm bottom diameter and 120 mm in height by an electric resistance furnace. The additions of an Al3Ti1B master alloy (in wt%) at concentrations of 50, 100, 200, 500 and 1000 ppm Ti were introduced into the molten melt at 720 ± 3 °C, which was then stirred and held for 5 minutes before being transferred to the UST platform. When UST was not applied the alloy was allowed to cool and solidify without the insertion of the sonotrode.

For the UST experiments the ultrasonic device consisted of a 2-kW commercial ultrasound generator, an air cooled 20-kHz piezoelectric transducer and a sonotrode made of a molybdenum alloy with an 18 mm diameter tip. When UST was applied to the same range of compositions as listed above, the ultrasonic sonotrode was switched-on without preheating and then immersed 15 mm below the top surface of the melt in the graphite-clay crucible. Two K-type thermocouples were inserted into the melt beside the sonotrode: one adjacent to the wall of the crucible and the other being placed 12.5 mm towards the centre of the crucible. Both thermocouples were positioned 45 mm above the bottom of the crucible. The UST experiments were conducted with a fixed power input of 1 kW at a peak-to-peak amplitude of 20 μm from 700 °C, which is 40 °C above the liquidus temperature, for 4 minutes during subsequent solidification, ceasing at 653 °C. Heat from the melt is absorbed by and transferred through the un-preheated sonotrode. The high superheat ensures the sonotrode is heated to a stable operating temperature before the melt reaches the liquidus temperature although it is expected that heat will continue to be absorbed by the sonotrode during subsequent cooling. At the applied amplitude of 20 μm, the ultrasonic intensity (*I*) level is about 975 w cm^−2^, estimated using Equation (4)^4^

4 $$I=\frac{1}{2}\,\rho c{(2\pi fA)}^{2}$$

in which $\rho$ is the density of molten Al (2375 kg m^−3^), *c* is the speed of sound in molten Al (≈1.3 × 10^3^ ms^−1^ ^4^), *f* is the frequency (20 kHz) and *A* is the amplitude. The estimated intensity level is far more than the threshold ultrasonic intensity required to produce cavitation in molten Al, which ~80 Wcm^−2^ ^4^.

The temperature data was collected by a data-acquisition system with a sampling rate of four readings per second. The cooling rate was 0.68–0.78 °C/sec without UST and 0.82–0.96 °C/s with UST from 20 °C above the liquidus. Macrographic samples were sectioned along the centre symmetrical axis of the solidified casting and mechanically ground and polished for observation. In order to measure the grain size, small metallographic samples were cut at 45 mm (the same height as the thermocouples and 25 mm below the sonotrode) from the bottom of the sectioned piece. Micrographs were obtained by a Leica Polyvar microscope with polarized light after anodizing using a 0.5% HBF_4_ solution for about 20 seconds at 30 V. The grain sizes were measured using the linear intercept method (ASTM E112–10).

## Summary

The addition of Al3Ti1B master alloy and the application of UST to CP Al from above the liquidus until the end of solidification both individually produced considerable grain refinement. Their combined action results in additional grain refinement. The relationship between grain size and grain density and the inverse growth restriction factor indicates that the application of UST and the increasing Ti content increase the nucleation efficiency. It is proposed that in addition to the possible effects of UST on wetting, deagglomeration, nucleation and fragmentation, acoustic streaming effectively distributes nucleated grains and lowers the temperature gradient in the melt which decreases the size of the nucleation-free zone thus increasing the number of activated TiB_2_ particles and survived aluminium fragments. All these factors lead to a reduction in grain size. Through comparison with an earlier similar study on an Al-2% Cu-based alloy, it appears the mechanisms of grain refinement are the same and that the data for CP Al and Al-2% Cu alloys are consistent once evaluated in terms of the growth restriction factor. A predictive grain size model was applied to take into account the changed thermal environment when UST is applied enabling more successful nucleation events to occur and survive. However, this model currently only accounts for a fraction of the additional nucleation events, indicating that additional nucleation probably occurs in the cavitation zone near the sonotrode surface and by a number of possible mechanisms such as wetting, deagglomeration and dendrite fragmentation. More research is required to be able to quantify the relative contribution of these mechanisms.
